# Remimazolam tosylate's long-term sedative properties in ICU patients on mechanical ventilation: effectiveness and safety

**DOI:** 10.1186/s40001-023-01440-9

**Published:** 2023-10-21

**Authors:** Zhiyuan Yao, Zhaomin Liao, Guang Li, Lu Wang, Liying Zhan, Wenfang Xia

**Affiliations:** Department of Critical Care Medicine, Renmin Hospital of Wuhan University, Wuhan, 430060 Hubei People’s Republic of China

**Keywords:** Remimazolam tosylate, Mechanical ventilation, Intensive care unit, Propofol, Midazolam, Sedation

## Abstract

**Objective:**

This study compared remimazolam tosylate with propofol or midazolam to assess its safety and effectiveness for long-term sedation of intensive care unit (ICU) patients requiring mechanical ventilation.

**Methods:**

Adult patients in the ICU receiving sedation and mechanical ventilation for longer than 24 h were included in this single-center, prospective, observational study. Depending on the sedatives they were given, they were split into two groups (midazolam or propofol group; remimazolam group). ICU mortality was the main result. Laboratory tests, adverse events, and the length of ICU stay were considered secondary outcomes.

**Results:**

A total of 106 patients were involved (46 received propofol or midazolam versus 60 received remimazolam). Age (*P* = 0.182), gender (*P* = 0.325), and the amount of time between being admitted to the ICU and receiving medication infusion (*P* = 0.770) did not substantially differ between the two groups. Multivariate analysis revealed no statistically significant difference in ICU mortality between the two groups. The remimazolam group showed less variability in heart rate (*P* = 0.0021), pH (*P* = 0.048), bicarbonate (*P* = 0.0133), lactate (*P* = 0.0002), arterial blood gas analyses, liver, and kidney function. The Richmond Agitation and Sedation Scale scores, length of ICU stay, and occurrence of adverse events did not exhibit significant differences between the two groups.

**Conclusion:**

Remimazolam tosylate did not increase the total inpatient cost, the incidence of adverse events, and ICU mortality in patients with mechanical ventilation. These findings suggest that remimazolam may represent a promising alternative for sedation in the ICU setting.

**Supplementary Information:**

The online version contains supplementary material available at 10.1186/s40001-023-01440-9.

## Introduction

In the intensive care unit (ICU), mechanical ventilation is a vital form of life support, but it also has several negative side effects [1, 2]. As a result, patients who get mechanical ventilation usually need sedative therapy [3]. Propofol and midazolam are currently used often in ICU sedation [4]. Propofol has a quick onset, a short half-life, no body buildup, a quick recovery, and fewer side effects [5]. Water-soluble benzodiazepine midazolam has a rapid onset, brief duration, no accumulation in the body, and a rapid metabolism [6]. It also has modest intravenous stimulation. However, the cardiovascular system is somewhat inhibited by both midazolam and propofol [5, 7]. Because both midazolam and propofol are metabolized by the liver and kidneys, prolonged administration is likely to result in drug accumulation [8, 9], which can increase the time needed for recovery and weaning in ICU patients who are on mechanical ventilation. Even though the two medications are frequently combined in clinical practice, side effects such as blood pressure drops and respiratory depression are inevitable [10].

Remimazolam is a benzodiazepine sedative with an ultra-short half-life that acts on γ-aminobutyric acid type A (GABAa) receptor receptors [11, 12]. In vivo, plasma esterase hydrolysis converts it into the inert molecule CNS 7054, and this process is not dependent on the liver or the kidneys. Remimazolam has a high clearance rate, a quick metabolism, and quick and predictable onset and clearance curves [11, 13]. According to earlier research [14–17], remimazolam has fewer side effects such as hypotension, injection discomfort, and respiratory depression after prolonged treatment. It also shows sufficient safety when used in patients with severe illness [18], and has certain value in reducing sepsis liver injury [19]. As a result, we think that remimazolam besylate is a secure and reliable sedative that can be used on ICU patients who are receiving mechanical ventilation. This study's objective was to compare remimazolam tosylate to propofol or midazolam to assess its effectiveness and safety in the long-term sedation of ICU patients undergoing mechanical ventilation.

## Methods

### Research design

This single-center, prospective, observational study compared the effectiveness and safety of remimazolam with propofol or midazolam for long-term sedation in patients on mechanical ventilation in the ICU. This study adhered to the Helsinki Declaration and was authorized by the Clinical Research Ethics Committee of Renmin Hospital of Wuhan University (WDRY2021-K008). Everyone who took part, or members of their close family, gave written informed consent. Before it began, this study was registered in the Chinese Clinical Trial Registry (ChiCTR2100051478). The study was carried out between October 2021 and December 2022.

### Participants

Age greater than or equal to 18 years, continuous use of mechanical ventilation and sedative use (midazolam, propofol or remimazolam) greater than or equal to 24 h are the inclusion criteria for this study. Exclusion criteria: (1) using two or more sedatives concurrently; (2) women who are pregnant or lactating; (3) patients allergic to benzodiazepines, opioids or propofol, or have contraindications; (4) patients with a history of drug use such as heroin, marijuana, and methamphetamine; (5) patients with positive serum ethanol test, with a history of alcoholism or alcohol dependence; (6) patients with mental illness; (7) patients with severe liver or kidney dysfunction (Kidney: Rifle criteria ≥ F; Liver: total plasma protein < 30 g/l; bilirubin > 85 mol/l) [20–22]; (8) hemodynamic instability: bradycardia (heart rate < 50 beats/min), hypotension (systolic blood pressure < 90 mmHg even with vasoactive drugs), second degree and above atrioventricular block and no pacemaker; (9) refusing to sign an informed consent form; (10) patients who have been included in other clinical studies.

### Procedures

The patients were divided into two groups (the remimazolam group and the propofol or midazolam group) depending on the sedative drugs they used (the researchers screened participants based on inclusion and exclusion criteria, selected sedative drugs based on the patient's vital signs, underlying diseases, and the thorough evaluation of the attending physician). The treatment plan of all enrolled patients was formulated by the responsible physician of each patient according to the patient's condition. During the study period, the patient's existing treatment plan was not interrupted, and we did not intervene in the patient’s treatment plan. All of the medications put to the test were continuously infused intravenously. The dosage schedule was as follows: (1) Remimazolam (Jiangsu Hengrui Pharmaceutical Co., Ltd.), the first dose was 0.2 mg/kg, and the maintenance dose was 0.16 mg/kg/h; (2) Propofol (Xi’an Libang Pharmaceutical Co., Ltd.), initial dose 2.0 mg/kg, maintenance dose 1.5 mg/kg/h; (3) Midazolam (Jiangsu Enhua Pharmaceutical Co., Ltd.), the first dose of 0.12 mg/kg, maintenance dose of 0.075 mg/kg/h. Analgesic regimen: (1) remifentanil, 4 ~ 9 μg/kg/h; (2) Nalbuphine hydrochloride, 1.0 ~ 1.4 mg/kg/h. Patients with mild to moderate liver and kidney dysfunction and elderly patients (age ≥ 60 years) were given a reduced infusion of midazolam and propofol based on the above doses. According to previous studies, the pharmacokinetics of remimazolam are not affected by liver and kidney function [23], so the dose of remimazolam is not adjusted. However, in the course of clinical use, doctors will pay close attention to the changes of liver and kidney function in patients. When the liver and kidney function deteriorate due to sedative drugs, the tested drugs will be discontinued or other sedative drugs will be replaced in time. In our study, titration was used to adjust the patient's sedation depth to maintain the Richmond Agitation and Sedation Score (RASS) score between − 4 and 0. At each dose adjustment, the adjustment range was not more than 0.1 mg/kg/h. The adjustment of sedative drug dose is mainly based on the RASS score. When the RASS score is within the target range, if the patient's respiration, blood pressure, heart rate, and pulse oxygen saturation change, the monitoring equipment itself and the patient's condition change should be considered first. After eliminating the above problems, it is considered to be related to sedatives. At this time, the sedative dose can be adjusted according to the patient's vital signs, but the sedation depth is required to be maintained within the target range. Extubation, discontinuation of the test drug by the treatment physician for more than 24 h, and leaving the ICU were considered as the cessation criteria, whichever occurred first. Until they were discharged from the ICU, all patients were monitored.

The patient withdrew mechanical ventilation using programmed weaning, as follows: (1) The first step is preparing a weaning assessment. (1) When Fraction of Inspired Oxygen(FiO_2_) ≤ 0.5, Peripheral Oxygen Saturation(S_P_O_2_) ≥ 95%; (2) Set Positive End-Expiratory Pressure(PEEP) ≤ 8 cm H_2_O; (3) Normal arterial blood gas analysis; (4) No dependence on pressor drugs; (5) Hemodynamic stability; (6) Consciousness judgment. If the above standards are met, the second step is carried out. (2) Step 2: Brief spontaneous breathing test, measuring rapid shallow breathing index(RSBI); (1)The ventilator was set to ‘Continuous Positive Airway Pressure(CPAP)’ mode, FiO_2_ ≤ 0.5, pressure support(PS) 5 ~ 8 cm H_2_O, PEEP ≤ 5 cm H_2_O; (2) Under this setting, mechanical ventilation was performed for 2 ~ 3 min, and the RSBI was measured. If RSBI < 105, the third step is carried out; otherwise, restore mechanical ventilation. (3) Step 3: Spontaneous breathing test. (1) The ventilator was set to ‘CPAP’ mode, set FiO_2_ ≤ 0.5, PS was 5 ~ 8 cm H_2_O, PEEP ≤ 5cm H_2_O. (2) Continuous 1 h of spontaneous breathing without interruption, that is, the spontaneous breathing test was successful. If the following conditions occur, the spontaneous breathing test is interrupted: (1) respiratory rate > 35 times/min, more than 5 min; (2) S_P_O_2_ < 95%, more than 3 min; (3) Heart rate increased by more than 20% compared with baseline; (4) Systolic blood pressure was higher than 160 mmHg or lower than 90 mmHg. If the Spontaneous breathing test is successful, take the fourth step; otherwise, restore mechanical ventilation. (4) Step 4: Weaning. The mechanical ventilation was stopped, and oxygen was given at 2–3 L/min through a tracheal intubation catheter after weaning. (5) Step 5: Extubation. If Arterial Oxygen Saturation(SaO_2_) ≥ 95% and arterial blood gas analysis were normal under the condition of 2–3 L/min oxygen supply through tracheal intubation catheter, the tracheal intubation was pulled out after sufficient sputum suction, and the nasal catheter or mask was given oxygen at 1–2 L/min[24].

### Outcomes

ICU mortality was the main result. Hemodynamics, arterial blood gas analysis, liver and kidney function, adverse events, sedation expenses, and length of ICU stay were the secondary outcomes. The arterial blood gas analysis, liver, and kidney function were recorded three times (at enrolment, 24 h, and 48 h), and the hemodynamics were recorded nine times (at enrollment, 2 min, 5 min, 10 min, 20 min, 1 h, 2 h, and 48 h). Adverse events included tachycardia (heart rate > 120 beats/min), bradycardia (heart rate < 50 beats/min), hypotension (systolic blood pressure < 80 mmHg or diastolic blood pressure < 50 mmHg), and hypertension (systolic blood pressure > 180 mmHg or diastolic blood pressure > 100 mmHg).

### Statistical analysis

A sample estimate wasn't done because there weren't any hypotheses. For statistical analysis, GraphPad Prism 9.0 (GraphPad Software, San Diego, CA, USA) and SPSS 26.0 (IBM SPSS Statistics, Armonk, NY) were used. Continuous data were reported as median (interquartile range, IQR) or mean ± standard deviation. The differences between groups were examined using the Mann–Whitney *U* test, analysis of variance, or Student's t-test. Frequency and proportion were utilized to convey categorical data, while chi-square or Fisher's exact tests were used to examine differences. Repeated measures analysis of variance was used to compare repeated measures data (such as hemodynamics, blood gas analysis, and laboratory tests). Confounding factors (such as age and analgesic use) were controlled by covariates during the analysis. The multivariate analysis employed the Cox Proportional-Hazards Model. A forward elimination method with *P*-removal = 0.1 was used to include all variables in the univariate models with *P* < 0.2 [25]. Statistics were judged significant at *P* < 0.05.

## Results

**Fig. 1:**
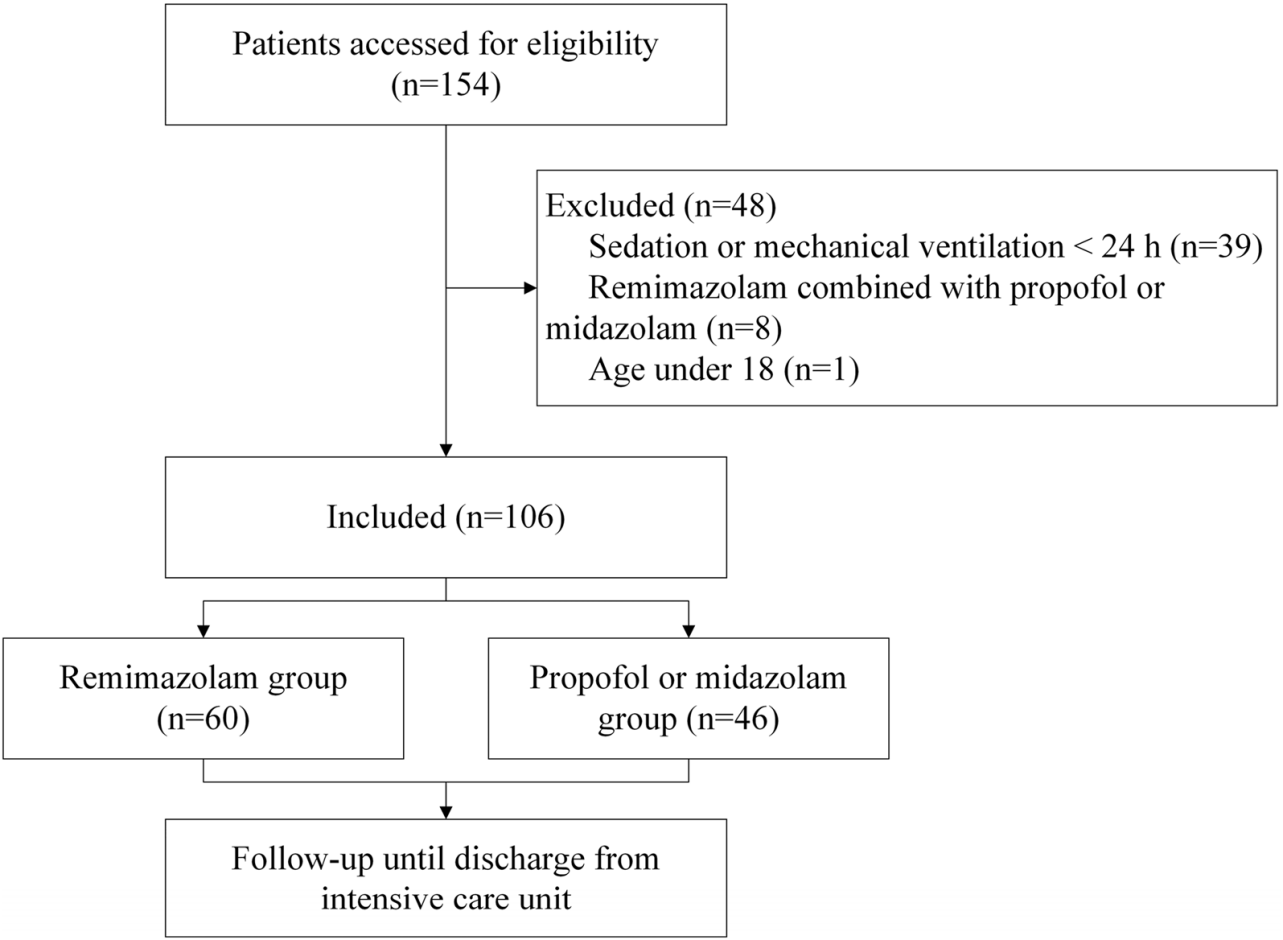
Flowchart of the study

106 individuals were eventually included in the research after 154 were initially assessed, including 60 in the remimazolam group and 46 in the propofol or midazolam group (Fig. 1). The patient's average age was 59.34 ± 14.7 years; 38 (35.6%) of them were beyond 65; and 70 (66.0%) of them were men. Basic traits including the Glasgow Coma Scale (GSC), the Acute Physiology and Chronic Health Evaluation II (APACHE II), common disorders, admission procedures, and the interval from ICU admission to the use of the test medicine did not significantly differ (Table 1).

**Table 1:** Baseline characteristics of the patients

Variables	Remimazolam (n = 60)	Propofol or midazolam (n = 46)	P-value
Age, years	61.0 ± 13.5	57.2 ± 16.1	0.182
Age group			0.542
< 65	37 (61.7)	31 (67.4)	
≥ 65	23 (38.3)	15 (32.6)	
Male	42 (70.0)	28 (60.9)	0.325
APACHE II score	22.0 (19.3, 27.0)	24.0 (22.0, 28.0)	0.175
Glasgow coma scale	5.0 (3.0, 9.8)	4.0 (3.0, 9.0)	0.083
Cerebral hemorrhage	17 (28.3)	14 (30.4)	0.814
Shock	15 (25.0)	8 (17.4)	0.346
Hepatic insufficiency	14 (23.3)	14 (30.4)	0.411
Renal insufficiency	14 (23.3)	8 (17.4)	0.455
Sepsis	5 (8.3)	2 (4.3)	0.696
Type of ICU admission			0.090
Other departments or medical institutions	32 (53.3)	32 (69.6)	
Emergency	28 (46.7)	14 (30.4)	
The time from admission to ICU to the use of the test drug, h	3.0 (0.0, 26.5)	6.0 (0.0, 22.0)	0.770

Data are n(%), mean ± SD or median (interquartile range); APACHE II = Acute Physiology and Chronic Health Evaluation II

Table 2 displays the duration of drug use and the use of analgesics. The median dose of midazolam was 3.3 (2.7, 6.6)mg/h, 31.7 (16.7, 71.1)mg/h for propofol, and 10.3 (7.3, 11.7)mg/h for remimazolam. There were no statistically significant differences in the two groups' drug use durations. Nalbuphine hydrochloride and remifentanil did not differ.

**Table 2:** Details of study drug administration

Variables	Remimazolam (*n* = 60)	Propofol or midazolam (*n* = 46)	*P*-value
Duration of drug use^a^, h	56.5 (45.0, 90.0)	59.0 (46.0, 110.0)	0.495
Remifentanil use	58 (96.7)	44 (95.7)	1.000
Dose of remifentanil, μg/h	407.0 (287.3, 483.7)	339.7 (236.8, 444.4)	0.106
Nalbuphine hydrochloride use	50 (83.3)	40 (87.0)	0.785
Dose of nalbuphine hydrochloride, mg/h	7.9 (4.8, 9.4)	6.8 (4.4, 10.3)	0.363

Data are *n*(%), mean ± SD or median (interquartile range)

^a^The time from the first application of the test drug to the standard of cessation

All-cause mortality was greater in the remimazolam group (36.7% vs. 17.4%, *P* = 0.032, Table 3). Because mortality is affected by many factors, we used a multivariate Cox proportional hazards model to study whether ICU mortality is related to the use of different sedatives. Firstly, the patient's gender, age, current medical history, grouping, patient source, APACHE II score, GCS score, and disease were included in the univariate Cox proportional hazards model. Then, according to the results of the univariate model, the factors with *P* < 0.2 were included in the multivariate model [25]. Based on clinical experience and previous research results, as well as the results of the univariate Cox proportional hazards model, we introduced variables such as grouping, age, gender, GCS score, hypertension, anemia, cerebral hemorrhage, shock, coagulation dysfunction, and surgery into the multivariate Cox proportional hazards model. Results showed that remimazolam did not, however, raise the probability of ICU death (Additional file 1: Table S1, *P* = 0.053). The RASS scores of the two groups attained the desired value during the observation period, and there was no significant difference between them (Table 3). The remimazolam group saw less mechanical breathing time overall (*P* = 0.038). Considering that there is a clear competitive relationship between mechanical ventilation and death, we further calculated the duration of mechanical ventilation in non-dead patients and also found that the duration of mechanical ventilation in the remimazolam group was shorter [57.00(40.75, 118.25) hours vs. 86.00(57.00, 132.00) hours, *P* = 0.047]. There was no obvious difference between the two groups in terms of the length of ICU and hospital stays. In the current study, tachycardia and hypotension predominated; adverse events associated with bradycardia did not occur. The frequency of adverse events did not significantly differ between the two groups. Remimazalam patients paid more on average for sedatives [RMB 907.3(685.3, 1500.6) yuan versus RMB 546.5 (308.2, 1250.4)yuan, *P* = 0.001]. However, we found that the total inpatient cost and ICU inpatient costs of patients in the remimazolam group were lower than those in the propofol or midazolam group.

**Table 3:** Outcomes

Variables	Remimazolam (n = 60)	Propofol or midazolam (*n* = 46)	*P*-value
RASS score	− 4.0 (− 4.0, -3.0)	− 4.0 (− 4.0, − 3.0)	0.317
Duration of mechanical ventilation, hours	75.0 (46.3, 158.8)	114.0 (70.0, 254.0)	0.038
All-cause ICU mortality	22 (36.7%)	8 (17.4%)	0.032
Length of ICU, days	6.0 (4.0, 10.0)	7.0 (4.0, 19.0)	0.450
Length of stay in hospital, days	10.0 (6.0, 24.8)	21.0 (9.0, 33.0)	0.061
Adverse events			
Tachycardia	18 (30.0)	16 (34.8)	0.601
Hypotension	11 (18.3)	8 (17.4)	1.000
Hypertension	3 (5.0)	4 (8.7)	0.464
Cost of sedative per case, yuan	907.3 (685.9, 1383.7)	546.5 (308.2, 1250.4)	0.001
Total Inpatient cost, yuan	136,806.50 (86,932.05, 223,212.88)	217,919.19 (91,237.08, 247,396.00)	0.189
ICU Inpatient Costs, yuan	101,399.74 (63,899.72, 191,501.65)	104,766.62 (57,557.64, 231,638.21)	0.684

Data are *n*(%), mean ± SD or median (interquartile range)

R*ASS*   Richmond agitation and sedation scale

Vital indicators including respiration rate and systolic and diastolic blood pressure were more stable in the remimazolam group during the observation period, but there was no statistically significant difference (Additional file 1: Figure S1). At 2–5 min after taking the drug, both patient groups displayed an increase in heart rate and a fall in blood pressure, which gradually reverted to the baseline level at which they were recruited. There was a statistically significant difference in heart rate between the two groups at the time of enrolment and 2 min after injection (*P* < 0.05), but there was no difference during long-term use. The heart rates of patients who received remimazolam were consistent over time, but those who received propofol or midazolam showed a significant downward trend with time (Additional file 1: Figure S1C, *P* = 0.0021), while all of the heart rates were still within the normal range.

The remimazolam group's arterial blood gas analysis results, including potassium ion, carbon dioxide partial pressure, and oxygen partial pressure during the observation time, were more stable, although there was no statistically significant difference (Additional file 1: Figure S2). In comparison to the propofol or midazolam groups, the remimazolam group's pH, carbon dioxide partial pressure, bicarbonate, and lactate changed less (Additional file 1: Figure S3A, D, F, all *P* < 0.05). During the observation period, there was no statistically significant difference between the two groups in terms of bilirubin, liver enzyme, albumin, urea, creatinine, and troponin (Additional file 1: Figure S3). The patients in the remimazolam group, it turned out, experienced less volatility in the aforementioned indices.

## Discussions

The results of this study's multivariate Cox proportional hazards model revealed that there was no statistically significant difference in ICU mortality between remimazolam tosylate, propofol or midazolam for patients receiving mechanical ventilation. There were no appreciable changes in terms of the Richmond Agitation and Sedation Scale, the length of ICU stay, or adverse events. The remimazolam group experienced mechanical ventilation for a shorter period. For ICU patients receiving mechanical ventilation, rimimazolam tosylate might be a promising sedative. The best sedatives for intensive care units should relieve the patient's stress, anxiety, and agitation while lessening the inhibition of fundamental physiological responses like the heart and lungs. Midazolam and propofol, two sedative medications frequently used in ICU, fall short of the standards. Remimazolam has been proven to be safe and effective in general anesthesia, postoperative sedation, and endoscopic treatment [14, 15, 17, 26, 27], but less research has been done on its safety and effectiveness in long-term sedation of ICU patients who are on mechanical ventilation.

The median infusion dose of remimazolam tosylate used in this trial was 10.1 (7.3, 11.7) mg/h, which is equivalent to the sedative effects of midazolam or propofol. The infusion dose of remimazolam in this study was lower than that in previous studies [28–30], mainly because there was no operational stimulation and most patients used two or more analgesics (such as remifentanil and nalbuphine hydrochloride), which may have increased the depth of sedation to some extent. Participants in previous studies were primarily undergoing endoscopic treatment or surgery in this study, however. Remimazalam is safe when used for mild to moderate sedation, according to prior research [31]. The outcomes of this investigation add to the evidence for the security of deep sedation. Because the patients in this study were critically ill (high APACHE II score, low GSC score), it’s possible that the RASS score was biased because the majority of the patients were in apathy.

Between the two groups, there was no discernible difference in ICU mortality or ICU stay time. This study's findings were in line with those of a randomized controlled study that found no appreciable difference between remimazolam and propofol in terms of 28 day mortality or the length of mechanical ventilation [31]. The sample sizes for both the trials mentioned above and this one are small, and the remimazolam group exhibited a greater risk of mortality from all causes. To more clearly define its link with ICU mortality, a study with a bigger sample size is required. During the observation period, changes in vital signs like systolic and diastolic blood pressure and respiratory rate did not differ significantly between the two groups, according to the study's findings. However, the indices above were more stable in the remimazolam group, particularly in the heart rate (*P* = 0.0021), indicating that remimazolam had less of an inhibitory effect on the cardiovascular system over time, which was consistent with previous research results [13]. We also noticed that the patient's blood pressure dropped while taking the test drug and that their heart rate rose in 2 to 5 min after taking it. Remimazolam's sedative onset time has been determined by prior research to be 1.5–2.5 min [14], however, it is nevertheless important to monitor patients' vital signs as soon as it is administered. The patient's hemodynamic indices eventually reached the level they had at the time of enrollment during a subsequent observation. Although there was no statistically significant difference between the propofol or midazolam groups in this trial, this was consistent with earlier studies [32]. Tachycardia and hypotension were the most frequent adverse events in the patients in the remimazolam group.

The arterial blood gas analysis, and liver, and kidney function were not significantly different between the two groups in this study, however, it is obvious that the aforesaid indicators of patients in the remimazolam group fluctuated less. Remimazolam is an ultra-short-acting sedative that does not rely on liver and renal metabolism, according to prior research [28, 33]. That it has little impact on liver and renal function was validated by this study. The sample sizes of the two patient groups are not exactly matched in this observational study and the results may not be reliable due to the small numbers in the propofol and midazolam groups. To support the aforementioned findings, larger samples or randomized controlled research are required.

This research has several restrictions. First off, this study has a tiny sample size and is a single-center observational study. Although the essential traits of patients can be compared well, there is not a 1:1 match, therefore, some judgments may be skewed. Second, no changes were made to the patient's initial treatment schedule during the course of the trial. After the observation period was over, some patients received further sedative medications; hence, some findings might not be accurate. Third, we cannot guarantee that every physician will adhere to the recommendations for the use of sedative drugs, which may result in inconsistent dosages of the study medication. Finally, additional consideration should be given to patient delirium in the future as this study did not evaluate other unfavorable occurrences like them.

## Conclusion

The results of this single-center, prospective, observational study showed that compared with propofol or midazolam, the long-term sedation of remimazolam tosylate for patients with mechanical ventilation in ICU did not increase the total inpatient cost, the incidence of adverse events, and ICU mortality. It indicates that remimazolam may be a promising alternative to ICU sedation. However, larger sample studies are needed to provide more accurate conclusions.

### Supplementary Information

**Additional file 1: Table S1.** Results of the analysis of the ICU mortality using a COX Proportional-Hazards Model. **Figure S1.** Hemodynamics of patients during observation period. * represents a statistically significant difference in univariate analysis between groups. *P*-value is the result of repeated measures analysis of variance. *P*<0.05 was considered statistically significant. **Figure S2.** Arterial blood gas analysis during the observation period. * represents a statistically significant difference in univariate analysis between groups. *P*-value is the result of repeated measures analysis of variance. *P*<0.05 was considered statistically significant. **Figure S3.** Liver and kidney function indexes and other laboratory test results of patients during observation period. * represents a statistically significant difference in univariate analysis between groups.

## Data Availability

A reasonable request should be made to the corresponding author for the datasets used and/or analyzed during this study.

## Abbreviations

APACHE II: Acute physiology and chronic health evaluation II
GABAa: γ-Aminobutyric acid type A
GSC: Glasgow coma scale
ICU: Intensive care unit
IQR: Interquartile range
RASS: Richmond agitation and sedation scale
