# Supplementary material for: Remimazolam tosylate's long-term sedative properties in ICU patients on mechanical ventilation: effectiveness and safety
Source: Eur J Med Res. 2023 Oct 21;28:452. doi: 10.1186/s40001-023-01440-9 (PMC10590506; doi:10.1186/s40001-023-01440-9)
Supplement: Supplementary file 1 — Additional file 1: Table S1. Results of the analysis of the ICU mortality using a COX Proportional-Hazards Model. Figure S1. Hemodynamics of patients during observation period. * represents a statistically significant difference in univariate analysis between groups. P-value is the result of repeated measures analysis of variance. P<0.05 was considered statistically significant. Figure S2. Arterial blood gas analysis during the observation period. * represents a statistically significant difference in univariate analysis between groups. P-value is the result of repeated measures analysis of variance. P<0.05 was considered statistically significant. Figure S3. Liver and kidney function indexes and other laboratory test results of patients during observation period. * represents a statistically significant difference in univariate analysis between groups. [file 40001_2023_1440_MOESM1_ESM.docx]

| Table S1 Results of the analysis of the ICU mortality using a COX regression analysis | | | | | | | |
| --- | --- | --- | --- | --- | --- | --- | --- |
| **Factor** | **Univariate analysis** | | |  | **Multivariate analysis** | | |
|  | **Coefficient** | **(95% CI)** | ***P*-value** |  | **Coefficient** | **(95% CI)** | ***P*-value** |
| Group (remimazolam) | 2.375 | (0.817, 6.903) | 0.112 |  |  |  | 0.053 |
| Age (years) | 0.978 | (0.954, 1.003) | 0.080 |  |  |  | 0.063 |
| Sex (male, %) | 2.494 | (0.926, 6.715) | 0.071 |  |  |  | 0.143 |
| APACHE II score | 1.032 | (0.951, 1.120) | 0.449 |  |  |  |  |
| Glasgow coma scale | 0.917 | (0.805, 1.043) | 0.188 |  |  |  | 0.210 |
| Hypertension | 1.997 | (0.886, 4.502) | 0.095 |  |  |  | 0.956 |
| Pulmonary infection | 1.329 | (0.613, 2.879) | 0.472 |  |  |  |  |
| Anemia | 0.064 | (0.008, 0.491) | 0.008 |  | 0.070 | (0.009, 0.542) | 0.011 |
| Cerebral hemorrhage | 3.017 | (1.319, 6.902) | 0.009 |  |  |  | 0.085 |
| Shock | 0.107 | (0.014, 0.790) | 0.028 |  | 0.123 | (0.017, 0.916) | 0.041 |
| Respiratory failure | 1.543 | (0.682, 3.490) | 0.298 |  |  |  |  |
| Renal insufficiency | 0.767 | (0.259, 2.267) | 0.631 |  |  |  |  |
| Hepatic insufficiency | 1.059 | (0.412, 2.719) | 0.906 |  |  |  |  |
| Metabolic disturbance of electrolyte | 1.354 | (0.566, 3.242) | 0.496 |  |  |  |  |
| Upper gastrointestinal hemorrhage | 0.663 | (0.154, 2.862) | 0.582 |  |  |  |  |
| Hypoproteinemia | 0.780 | (0.278, 2.192) | 0.637 |  |  |  |  |
| Coagulation disorders | 0.118 | (0.015, 0.926) | 0.042 |  |  |  | 0.727 |
| Diabetes | 0.833 | (0.248, 2.802) | 0.768 |  |  |  |  |
| Sepsis | 0.415 | (0.056, 3.089) | 0.391 |  |  |  |  |
| Type of ICU admission (emergency) | 1.664 | (0.740, 3.741) | 0.218 |  |  |  |  |
| Operation | 0.316 | (0.123, 0.809) | 0.016 |  |  |  | 0.082 |
| Time from ICU admission to drug initiation, h | 0.997 | (0.990, 1.004) | 0.356 |  |  |  |  |
| APACHE II=Acute Physiology and Chronic Health Evaluation II | | | | | | | |


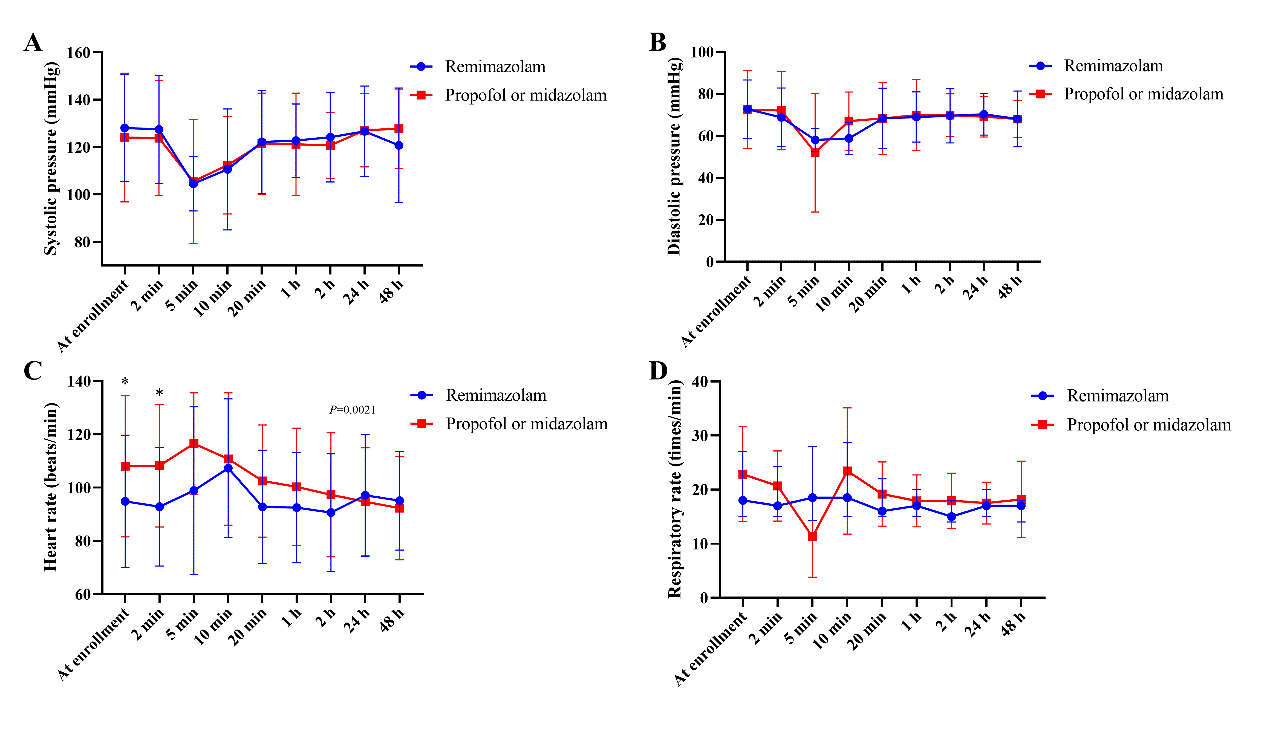


**Figure S1** Hemodynamics of patients during observation period. * represents a statistically significant difference in univariate analysis between groups. *P* value is the result of repeated measures analysis of variance. *P*<0.05 was considered statistically significant.


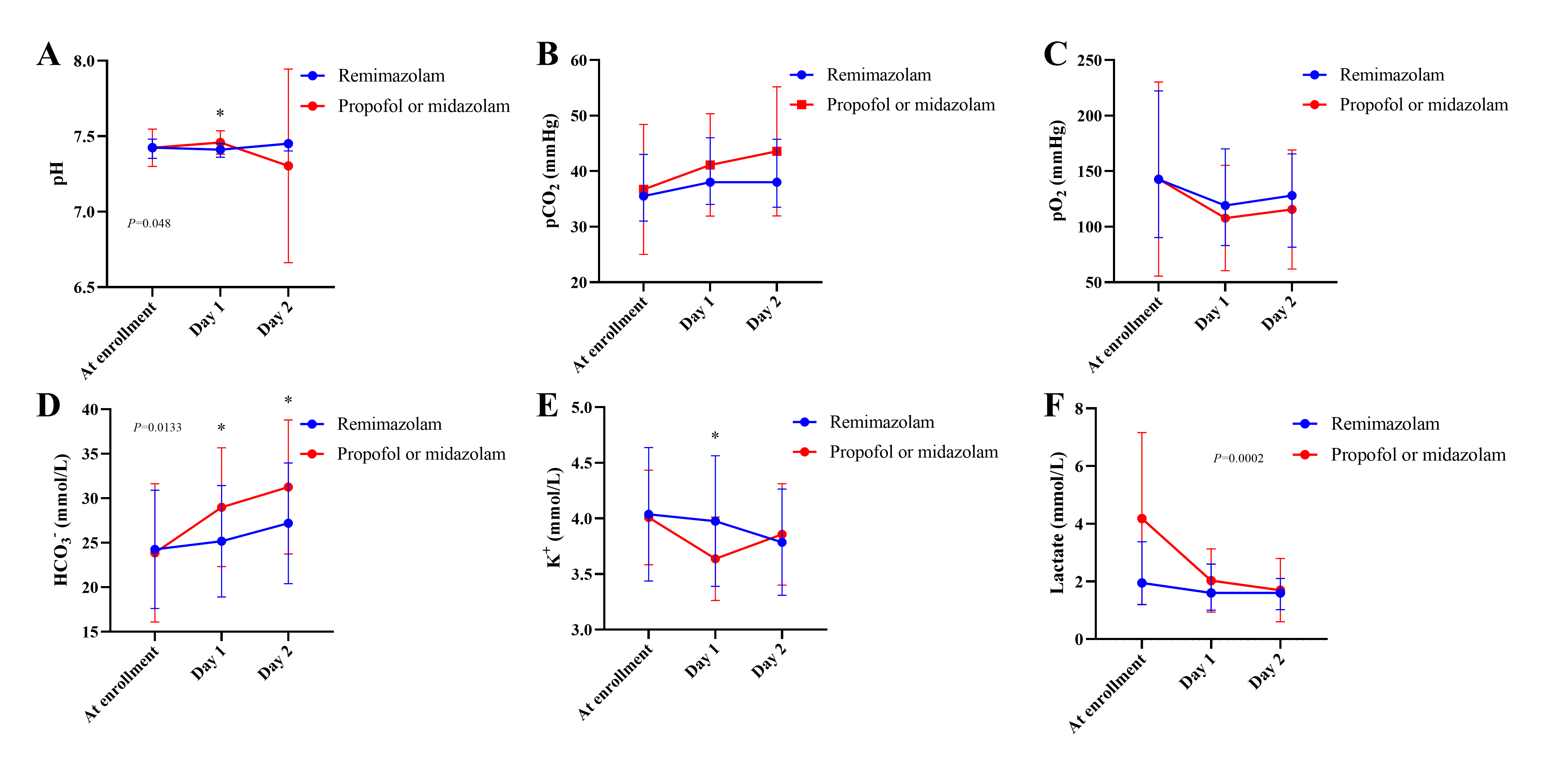


**Figure S2** Arterial blood gas analysis during the observation period. * represents a statistically significant difference in univariate analysis between groups. *P* value is the result of repeated measures analysis of variance. *P*<0.05 was considered statistically significant.


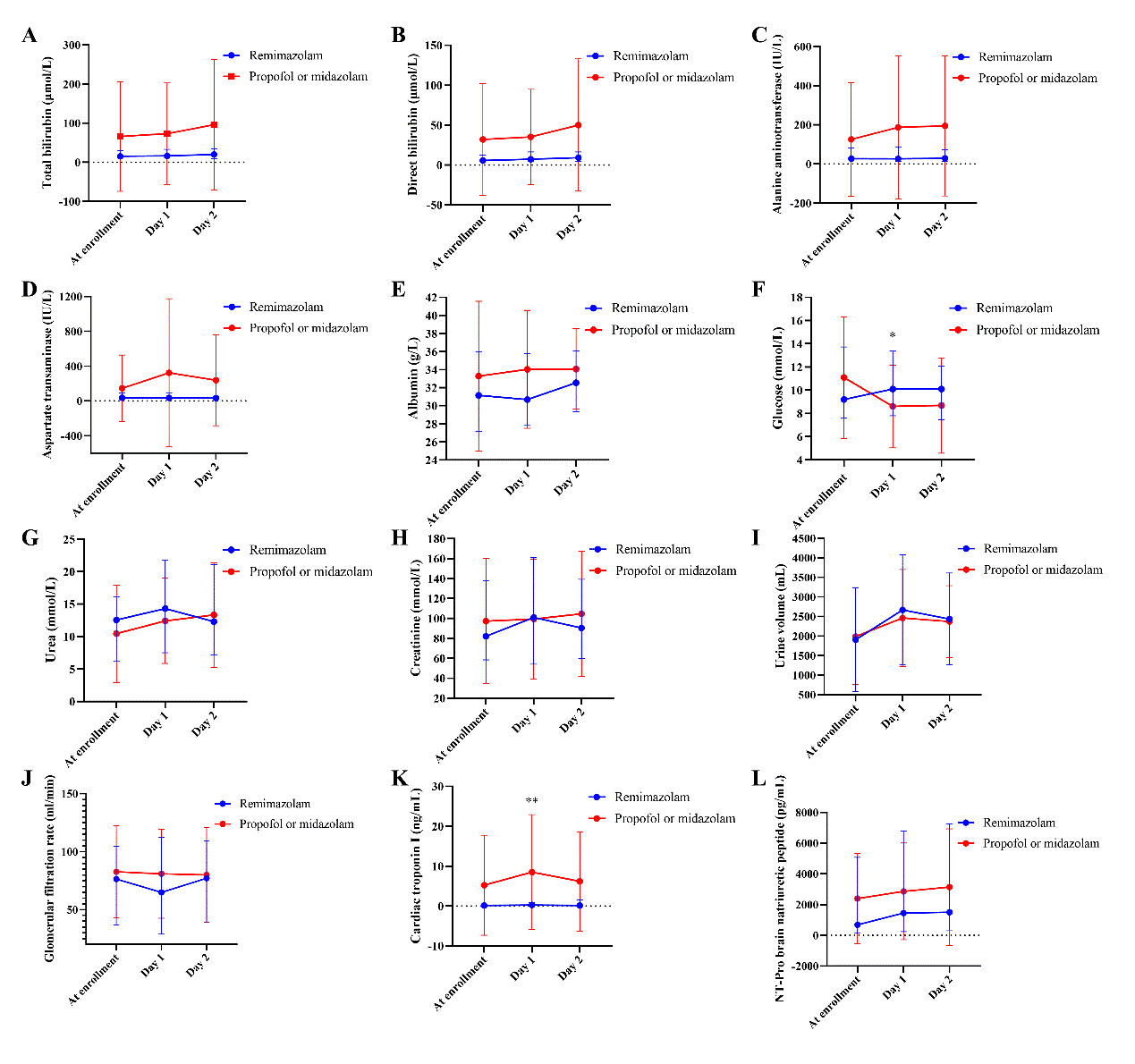


**Figure S3** Liver and kidney function indexes and other laboratory test results of patients during observation period. * represents a statistically significant difference in univariate analysis between groups.
